# Poplar protease inhibitor expression differs in an herbivore specific manner

**DOI:** 10.1186/s12870-021-02936-4

**Published:** 2021-04-09

**Authors:** Franziska Eberl, Thomas Fabisch, Katrin Luck, Tobias G. Köllner, Heiko Vogel, Jonathan Gershenzon, Sybille B. Unsicker

**Affiliations:** 1 Department of Biochemistry, Max Planck Institute for Chemical Ecology (MPI-CE), Hans-Knöll-Str. 8, 07745 Jena, Germany; 2 Department of Entomology, MPI-CE, Hans-Knöll-Str. 8, 07745 Jena, Germany

**Keywords:** Kunitz-type trypsin inhibitors; herbivore specificity; woody plants; tree defenses, Lepidoptera, Coleoptera, Salicaceae, Induced defenses, Proteinase inhibitors

## Abstract

**Background:**

Protease inhibitors are defense proteins widely distributed in the plant kingdom. By reducing the activity of digestive enzymes in insect guts, they reduce the availability of nutrients and thus impair the growth and development of the attacking herbivore. One well-characterized class of protease inhibitors are Kunitz-type trypsin inhibitors (KTIs), which have been described in various plant species, including *Populus spp*. Long-lived woody perennials like poplar trees encounter a huge diversity of herbivores, but the specificity of tree defenses towards different herbivore species is hardly studied. We therefore aimed to investigate the induction of KTIs in black poplar (*P. nigra*) leaves upon herbivory by three different chewing herbivores, *Lymantria dispar* and *Amata mogadorensis* caterpillars, and *Phratora vulgatissima* beetles.

**Results:**

We identified and generated full-length cDNA sequences of 17 *KTIs* that are upregulated upon herbivory in black poplar leaves, and analyzed the expression patterns of the eight most up-regulated *KTI*s via qRT-PCR. We found that beetles elicited higher transcriptional induction of *KTI*s than caterpillars, and that both caterpillar species induced similar *KTI* expression levels. Furthermore, *KTI* expression strongly correlated with the trypsin-inhibiting activity in the herbivore-damaged leaves, but was not dependent on damage severity, i.e. leaf area loss, for most of the genes.

**Conclusions:**

We conclude that the induction of KTIs in black poplar is controlled at the transcriptional level in a threshold-based manner and is strongly influenced by the species identity of the herbivore. However, the underlying molecular mechanisms and ecological consequences of these patterns remain to be investigated.

**Supplementary Information:**

The online version contains supplementary material available at 10.1186/s12870-021-02936-4.

## Background

Over millions of years plants have developed numerous strategies to defend themselves against plant-feeding animals. Apart from indirect defenses, which involve the recruitment of an herbivore’s natural enemies, plants can harm their attackers directly by producing mechanical barriers, chemical toxins and deterrents, or by using biochemical defenses that interfere with the herbivore’s enzymatic machinery. Among chemical defenses, most emphasis has been placed on low molecular weight metabolites, but defensive proteins exist, such as protease inhibitors (PIs) that reduce the digestibility of plant tissue for the feeding herbivore. By inhibiting proteolytic enzymes in the midgut of the herbivore, PIs diminish protein digestion and hence lower the availability of free amino acids required for herbivore growth and development [15]. The PIs found in plants are numerous and diverse, with 99 different inhibitor families currently described [32]. Those PI families, as well as distinct members within a family, vary in their activity towards the four types of proteases found in herbivore guts, namely serine -, cysteine -, aspartic acid -, and metalloproteases. In herbivorous insects, the most abundant protein-degrading enzymes are the serine proteases [15]. It is therefore not surprising that serine PIs are widely distributed in the plant kingdom [20, 21]. One of the best characterized classes of serine PIs are the Kunitz-type trypsin inhibitors (KTIs; also Kunitz-type protease inhibitors, KPI), of which some are also able to inhibit cysteine proteases [2, 6]. KTIs are relatively small proteins with a mass of 20 to 25 kDa [39], with a β-trefoil structure, consisting of a β-barrel and several loops, of which one is binding to the active site of the target protease [42]. The biological activity of KTIs has been demonstrated by using gut extracts in in vitro assays [16, 29], as well as monitoring the fitness of herbivores feeding on KTI-enriched diets [2, 6, 22, 25, 26, 30]. Since the first description of a KTI in soybean [19, 24], most subsequent studies have also focused on KTIs from legume species [16, 17, 22, 30, 36, 43]. However, KTIs in trees have gained more attention in past years. In species of the genus *Populus,* several KTIs have been identified and characterized [7, 27, 29, 37, 39], and some shown to be inducible by mechanical wounding or insect herbivory [27–29, 39]. For example, feeding by the forest tent caterpillar, a generalist herbivore, increased *KTI* transcript abundance locally and systemically in hybrid poplar leaves [28]. In fact, genes encoding for KTIs belong to the most up-regulated ones in systemic poplar leaves upon mechanical wounding [9]. In a study by Philippe et al. [39] it was shown that the transcriptional induction triggered by wounding varies among the KTIs and in a time-dependent manner. So far, most studies used *Malacosoma disstria*, a generalist lepidopteran species, to investigate herbivore-triggered KTI responses in poplar [27, 28, 39]. To our knowledge, the specificity of poplar KTI induction towards other herbivore species has not yet been investigated.

Specificity of response to different herbivores may be especially important for large, long-lived woody perennials like trees, which encounter a vast diversity of herbivores in their lifetimes. For example, it is well known that plants react differently to leaf-chewing herbivores than herbivores feeding on phloem-sap [13, 23]. Specificity of anti-herbivore defenses can also be observed within the same feeding guild, and even within the same species depending on the insect’s developmental stage. For example, early instar generalist caterpillars induced a stronger defense reaction in black poplar leaves than late instar caterpillars of the same species [31]. The underlying mechanism might be explained by HAMPs or DAMPS (herbivore- or damage-associated molecular patterns, respectively) that plants perceive when being attacked [13]. These are influenced by the physical attributes of herbivory, such as leaf area removal or the timing of tissue damage, but also by chemical cues such as salivary compounds of the herbivores [33]. All of these traits can be herbivore species-specific and may allow plants to distinguish among attackers and mount adequate and effective defenses against specific herbivores. In black poplar trees, such herbivore-specific reactions could be shown for signaling molecules [14], as well as chemical defense traits such as volatile emission [14, 31, 47]. In a recent study by Fabisch et al. [14], total PI activity against trypsin was more strongly induced by beetle feeding than by caterpillar feeding on black poplar leaves. However, to date, we do not know which specific genes are responsible for the observed differences in PI activity and whether or not transcription of PI-encoding genes differs between beetle- and caterpillar-fed leaves.

**Fig. 1:**
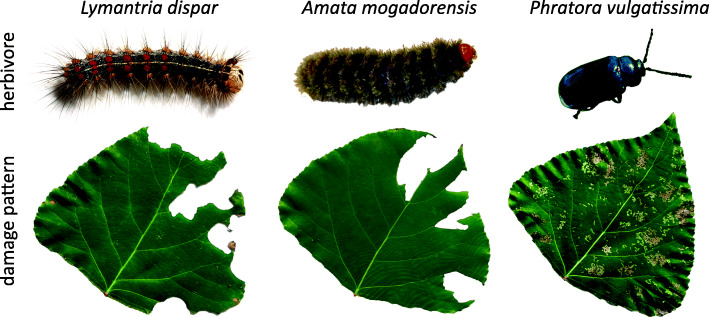
Insects used in this study and their damage pattern after 2 d feeding on black poplar leaves. *Amata mogadorensis* and *Lymantria dispar* (gypsy moth) remove large areas from the leaves, whereas *Phratora vulgatissima* (blue willow beetle) causes small, but numerous lesions

In this study, we therefore tested the hypothesis that different herbivore species induce *KTI* genes in a species-specific manner. We identified 17 *KTI* genes from a transcriptome of black poplar and generated full-length cDNA sequences of the most up-regulated ones. Gene expression patterns of these *KTI* genes as determined by qRT-PCR upon herbivory by three different insect species (Fig. 1) show striking differences among the species.

## Methods

### Plants and insects

*Populus nigra* L. (Salicaceae) trees were grown from cuttings obtained from trees in a common garden near Jena, Germany. These trees were originally derived from a single female genotype from a *P. nigra* population (species identified by Sybille Unsicker based on morphological features) located in Küstrin-Kietz, Germany (52°34′1“ N, 14°38’3” E). Since cuttings for this study were taken from trees in a common garden, no permission was necessary for collecting plant material; a voucher specimen will be deposited in spring 2021 in the Herbarium Haussknecht (JE) in Jena, Germany. The cuttings were potted in 2 L pots, grown in the greenhouse (18/20 °C, night/day, relative humidity 60%, natural light with 9–14 h photoperiod, supplemented light for 12 h) and transferred to a climate chamber (18/20 °C, night/day; relative humidity 60%; photoperiod 16 h) 2 days before the onset of the experiment. Trees were either grown for 4 months to approximately 0.5 m (*Transcriptome samples*) or grown to a height of 1.6 m (approximately 6 months) and pruned back to 0.8 m 4 weeks before treatment (*Gene expression samples*).

*Lymantria dispar* L. (Erebidae, Lepidoptera) caterpillars are generalist feeders with a broad host range, preferably deciduous trees. *L. dispar* caterpillars were hatched from eggs kindly provided by the US Department of agriculture (USDA, Buzzards Bay, MA, USA) and reared on artificial diet (MP Biomedicals LLC, Illkirch, France) in a climate chamber (14/10 h, light/dark, 20–23 °C, relative humidity 60%) until they reached the third instar, the stage used for the experiments. This species is reared continuously at the MPI-CE.

*Amata mogadorensis* Blachier (Erebidae, Lepidoptera) caterpillars are also generalists with a preference for woody plants and shrubs*. A. mogadorensis* caterpillars were hatched from eggs provided by a private breeder (www.entomologenportal.de) and reared on black poplar foliage until they reached the third instar, the stage used for the experiment. Individuals were reared until adult stage to confirm the species identity.

*Phratora vulgatissima* L. (Chrysomelidae, Coleoptera) beetles are specialists, feeding on a narrow range of hosts within the Salicaceae. Beetles (taxonomically determined by Lars Möckel; individuals in alcohol available at the MPI-CE) were reared in the laboratory on black poplar trees.

### Experimental designs and sampling

Plant material from two different experiments was used to analyze the transcriptome (see *Transcriptome samples*) or the gene expression of Kunitz-type trypsin inhibitors (KTIs; see *Gene expression samples*).

#### Transcriptome samples

A leaf pool (8 leaves from the stem of a young black poplar tree (*n* = 4) was wrapped with gauze and then infested with *L. dispar* caterpillars (4 individuals per tree), adult *P. vulgatissima* beetles (6 individuals per tree), or left untreated (control). Due to time differences in the availability of the experimental insects, the beetle treatment was conducted two weeks earlier than the caterpillar treatment; both treatments had their own respective control group (*n* = 4), which was treated and sampled at the same time as the herbivore-treated plants, but was not exposed to herbivores. After 2 d, the treated leaves were flash-frozen in liquid nitrogen and stored at − 80 °C.

#### Gene expression samples

For gene expression analysis by qRT-PCR, leaf material from an experiment described in Fabisch et al. [14] was used, where further details on the methods are described. In short, a leaf pool (5 leaves) of black poplar trees (*n* = 10, but a random selection of 6 was used for gene expression analysis) was wrapped with PET bags (Bratschlauch, Toppits, Minden, Germany) and then infested with *L. dispar* caterpillars (10 per tree), *A. mogadorensis* (10 per tree), *P. vulgatissima* beetles (50 per tree), or left untreated (control). After 1 d, the number of caterpillars was reduced to prevent excessive leaf loss. After a total feeding period of 2 d, the leaves were photographed to assess the damage and subsequently flash-frozen in liquid nitrogen and stored at − 80 °C. The damage was quantified as leaf area loss from the photographs by reconstructing the original leaf area in the picture and counting the number of pixels representing the total and the removed leaf areas (Photoshop, Version 15.0.0, Adobe Systems Incorporated, San Francisco, USA). Pixels were converted to area (cm^2^) using a reference field in the photograph.

### RNA isolation and cDNA synthesis

Frozen leaves were ground in liquid nitrogen and RNA was isolated using the InviTrap Spin Plant Mini Kit (Stratec Biomedical AG, Birkenfeld, Germany), including DNase digestion. RNA concentration was measured with a NanoDrop 2000c spectrophotometer (Peqlab Biotechnologie GmbH, Erlangen, Germany). For transcriptome samples, an additional quality check was conducted with the RNA 6000 Nano Kit on a Bioanalyzer (Agilent, Santa Clara, CA, USA). cDNA was synthesized from RNA using SuperScript-III reverse transcriptase and oligo-dT primers (Thermo Fisher Scientific, Waltham, MA, USA).

### Transcriptome analysis

Sequencing was done at the Max Planck-Genome-Center (Köln, Germany) on a HiSeq 2500 (Illumina, San Diego, CA, USA) with 9 Mio reads per sample. Detailed information on quality control measures, the assembly of the de novo transcriptome and the annotation can be found in Eberl et al. [12], but the most relevant information will be summarized here. The annotation was done using, among others, BLAST, Gene Ontology (GO) and InterPro terms (InterProScan, EBI). Contigs encoding for potential KTI proteins were identified based on a positive BLAST hit against a known KTI in the NCBI nr database, GO terms associated with serine proteinase inhibitors and/or a hit against the Pfam domain PF00197 (Kunitz STI protease inhibitor), or InterPro domains IPR011065 (Kunitz inhibitor STI-like superfamily) and IPR002160 (Proteinase inhibitor I3, Kunitz legume). In order to identify further KTI candidates, the *P. nigra* transcriptome was uploaded in an internal database and used for BLAST analysis of poplar KTI sequences from NCBI (www.ncbi.nlm.nih.gov/) and Phytozome (https://phytozome.jgi.doe.gov/pz/portal.html). Digital gene expression analysis was carried out using CLC Genomics Workbench v9.1 to generate BAM (mapping) files, and expression levels were then estimated using QSeq Software (DNAStar Inc., Madison, WI, United States). The log2 (RPKM) values (normalized mapped read values; geometric means of the biological replicate samples) were used to calculate fold-change values. Differentially expressed genes were identified using the Student’s t-test (as implemented in Qseq) corrected for multiple testing using the Benjamini–Hochberg procedure to check the false discovery rate (FDR). With an FDR-corrected *p*-value less than 0.05 a gene was considered significantly differentially expressed.

In addition to the *KTI* gene sequences in the transcriptome of the herein described experiment, another *KTI* gene (*PnKTI B1*) was identified from an additional leaf transcriptome from the same *P. nigra* genotype and comparable *L. dispar* herbivory treatment (unpublished). Furthermore, another sequence encoding a KTI (*PnKTI A4*, or SQ33325–2), which was not present in the transcriptome, was identified during amplification from cDNA (see below) with primers originally designed for *PnKTI A13* (SQ33325).

### Cloning and sequencing of PI genes

Full-length open reading frames (ORF) were amplified from a mix of cDNA originating from herbivore-induced samples in a PCR using Phusion High Fidelity polymerase in HF-buffer according to the manufacturer’s manual (New England Biolabs GmbH, Frankfurt/Main, Germany). Primers were designed based on the putative ORF from the transcriptome whenever available, or with the ORF of the homologous genes retrieved from the NCBI data base (https://www.ncbi.nlm.nih.gov/). PCR products were cloned into a PCR4-blunt TOPO vector (Thermo Fisher) and fully sequenced using the Sanger protocol and capillary sequencing with an ABI Prism-Gene- Analyser 3130xl (Applied Biosystems).

### Sequence alignments and phylogenetic analysis

Homologs of *P. nigra KTI* sequences were identified using the BLAST-search of the NCBI data base (https://blast.ncbi.nlm.nih.gov/Blast.cgi) and the *P. trichocarpa* genome v3.0 (https://phytozome.jgi.doe.gov/). Alignments and similarity calculations were done with Geneious software (Biomatters, Auckland, New Zealand).

An amino acid alignment of poplar KTI proteins was constructed using the MUSCLE algorithm implemented in MEGA6 [46]. Tree reconstruction was done with MEGA6 using the Neighbor-Joining method and the JTT matrix-based method. All positions with less than 80% site coverage were eliminated.

### Gene expression analysis by qRT-PCR

cDNA (diluted 1:3 with water) from the *Gene expression samples* was used for quantitative real-time PCR (qRT-PCR), which was performed in a Brilliant III Ultra-Fast SYBR reaction mixture (Agilent) on a CFX Connect Real-Time PCR Detection System (Bio-Rad Laboratories, Hercules, CA, USA) with 40 2-step cycles (95 °C, 30s + 60 °C, 30s) and a melting curve from 53 to 95 °C. Primer sequences can be found in Table S2. The PCR products were verified by cloning and sequencing as described above. Gene expression was calculated using CFX Manager 3.1 (Bio-Rad) using the ΔΔc_q_ method and taking primer efficiencies into account. Values were normalized to *Actin* as a reference gene [41] and expressed relative to a control sample.

### Trypsin-inhibiting activity assay

In order to correlate gene expression with protease inhibitor activity, the trypsin-inhibiting activity assay was performed as described in Fabisch et al. [14]. In short, 10 mg freeze-dried leaf material was extracted with 400 μL buffer (25 mM Hepes-KOH, pH 7.2, 3% PVPP, 2% PVP, 1 mM EDTA) and the extract tested for trypsin-inhibiting activity in a colorimetric (cleavage of N-acetyl-DL-phenylalanine beta-naphthyl ester) in-gel diffusion assay.

### Statistical analysis

All data were checked for statistical assumptions, i.e. homogeneity of variances and normal distribution. Gene expression data for all *KTI* genes had to be log_10_-transformed to meet the statistical assumptions for parametric testing. For gene expression data, a one-way MANOVA (multivariate analysis of variance) coupled to a Tukey’s post-hoc test was applied. All statistical analyzes were conducted using SPSS 17.0 (SPSS, Chicago, IL, USA).

## Results

### Identification of herbivore-induced Kunitz-type trypsin inhibitors

**Fig. 2:**
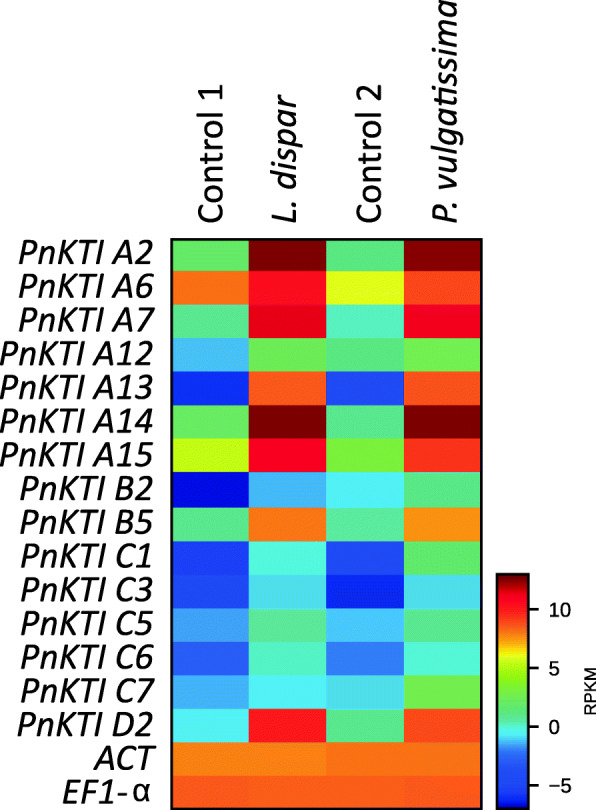
Expression of Kunitz-type trypsin inhibitor (*KTI*) genes in black poplar leaves after feeding by gypsy moth caterpillars (*L. dispar*) or blue willow beetles (*P. vulgatissima*) compared to their respective controls (Control 1 and 2), and compared to *actin* (*ACT*) and *elongation factor 1-α* (*EF1-α*) as constitutively expressed ‘house-keeping genes’. Shown are the mean RPKM (reads per kilobase of transcript per million mapped reads; *n* = 4) as result of the transcriptome analysis

The transcriptome of black poplar leaves with and without herbivory by two different insect species, *Lymantria dispar* (Lepidoptera) and *Phratora vulgatissima* (Coleoptera), was used to identify genes encoding herbivore-induced Kunitz-type trypsin inhibitors (KTIs). Among all sequences in the transcriptome, 45 were identified as protease inhibitor genes (PIs), of which 30 were up-regulated upon both caterpillar and beetle herbivory, seven showed different regulation patterns depending on herbivore identity, and eight were down-regulated upon herbivory by either of the herbivores (Table S3). Among the 45 PI genes, 15 belong to the *KTI*s, and were all up-regulated upon herbivory (Fig. 2). These 15 *KTI* sequences, plus two additionally identified *KTI* genes, were compared to previously described poplar *KTI*s (Table S4) and named according to the nomenclature of Ma et al. [27].

**Fig. 3:**
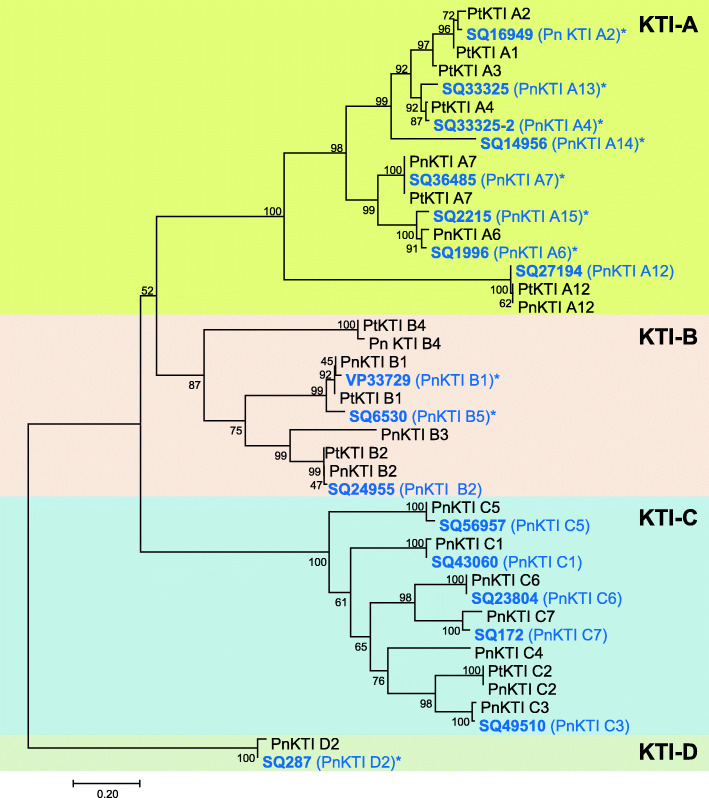
Phylogenetic tree of poplar KTI proteins. PnKTIs identified in this study are shown in blue and asterisks mark full-length cDNA sequences. The tree was inferred using the Neighbor-Joining method and the JTT matrix-based method. Bootstrap values (*n* = 500) are shown next to each node. The tree is drawn to scale, with branch lengths in the units of the number of amino acid substitutions per site

A phylogenetic analysis based on the amino acid alignment revealed that the KTIs cluster into 4 subfamilies (Fig. 3). Most of the 17 KTIs belong to the subfamilies A and C, whereas only one protein belongs to subfamily D. Interestingly, all members of the C-subfamily showed a low expression and were only marginally up-regulated upon herbivory in comparison to members of the other three subfamilies (Fig. 2). Therefore, KTIs from the subfamily C were not considered in further analysis. Out of the remaining *KTI* genes, those with the highest expression levels in herbivore-induced samples were chosen for cDNA sequencing, yielding the full-length open reading frames of ten *PnKTI* genes (Fig. 3).

### Herbivore-specific induction of KTI gene expression

To study the specificity of *KTI* gene expression, we used three different herbivore species that exhibit either similar (*L. dispar, Amata mogadorensis*) or different (*P. vulgatissima*) damage patterns on black poplar leaves (Fig. 1), but all cause similar leaf area loss (Table S1). In a previous study, we showed that total trypsin inhibitor activity in black poplar leaves is induced upon herbivory by three different herbivores, especially by *P. vulgatissima* [14]. To study this phenomenon at the transcriptional level, the relative gene expression of nine candidate *PnKTI*s was analyzed by qRT-PCR, using randomly selected samples from this previous study.

**Fig. 4:**
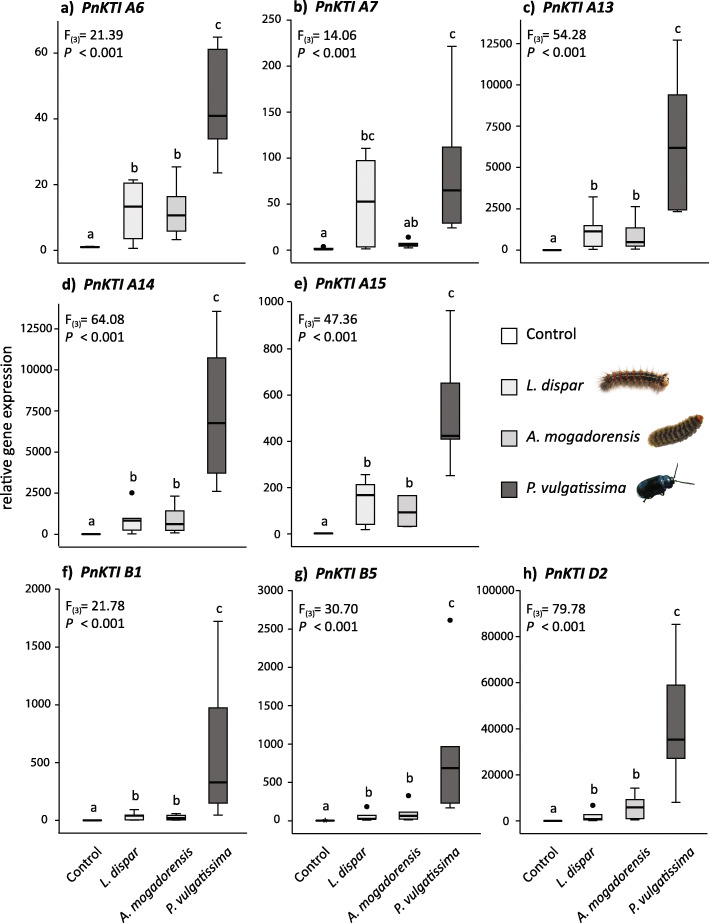
Transcript accumulation of Kunitz-type trypsin inhibitor genes (*KTI*s) of the **a**, **b**, and **d** subfamily in black poplar leaves after herbivory by two caterpillar species (*L. dispar, A. mogadorensis*) and one beetle species (*P. vulgatissima*). Shown are the gene expression normalized to *Actin* and relative to a control sample as boxplots (median with upper and lower quartile as bars; *n* = 6); results of the ANOVA are given in each graph. Different letters indicate significant differences among groups (*P* < 0.05; Tukey’s post-hoc test)

While *PnKTI A2* could not be amplified in the qPCR reaction and was therefore excluded from further analysis, all of the remaining eight *PnKTI* genes showed significant up-regulation upon herbivory by all of the tested insects (Fig. 4). A multivariate analysis including damage severity as covariate revealed that the herbivory treatment had the strongest effect on *PnKTI* gene expression (F_(24)_ = 7.230; *P* < 0.001).

Constitutive expression levels in undamaged leaves differed among the *PnKTI* genes, with members of the A subfamily generally displaying higher expression levels than those of the B and D subfamilies (Table S5). Upon herbivory, however, the genes showed even more apparent differences in their inducibility (Fig. 4). Caterpillar herbivory by *L. dispar* and *A. mogadorensis* resulted in an up-regulation of all *KTI* genes by approximately 10 (*PnKTI A6*) to 2000-fold (*PnKTI D2*) in comparison to the constitutive levels. All *KTI* genes were induced to similar levels by these two lepidopteran herbivores. Beetle herbivory by *P. vulgatissima,* however, caused a much stronger induction of *KTI* gene expression than caterpillar herbivory. Expression levels in beetle-damaged leaves increased up to 40,000-fold (*PnKTI D2*) compared to undamaged controls. Nevertheless, the induction levels differed substantially among the individual genes, ranging from approximately 40 (*PnKTI A6*) and several hundred (*PnKTI A15*, *B1*, *B5*) up to several thousand-fold (*PnKTI A13*, *A14*, *D2*). Interestingly, *PnKTI D2*, a gene with one of the lowest constitutive expression levels, showed by far the strongest relative induction upon both caterpillar and beetle herbivory (Fig. 4h).

When considering herbivore treatments only (excluding the undamaged control group), we found that the damage severity (% leaf area loss) did not have a substantial effect on expression levels of most of the *PnKTI*s. Only two genes, *PnKTI A7* and *PnKTI B1*, were significantly influenced by this factor in their expression (ANCOVA; *PnKTI A7*: F_(1)_ = 9.348, *P* = 0.009; *PnKTI B1*: F_(1)_ = 5.012; *P* = 0.042). Accordingly, the expression of the *PnKTI*s also did not correlate with the damage severity, except for *PnKTI A7*, which showed a positive relationship with leaf area loss (Spearman’s rank correlation: ρ = 0.556, *P* = 0.017). The total trypsin-inhibiting activity (Table S1 [14];), on the other hand, strongly correlated with the expression of all *PnKTI*s with a positive relationship (Table 1).

**Table 1:** Correlations of individual *PnKTI* gene expression versus total foliar trypsin-inhibiting activity (μg g^− 1^ DW; data from [14]) in all herbivore-treated (*L. dispar, A. mogadorensis,* and *P. vulgatissima* feeding) samples of black poplar leaves. Spearman rank-correlation, significant values are highlighted in bold font

***PnKTI***	Spearman’s ρ	***P***
*PnKTIA6*	0.707	**0.001**
*PnKTI A7*	0.648	**0.004**
*PnKTI A13*	0.646	**0.004**
*PnKTI A14*	0.730	**0.001**
*PnKTI A15*	0.700	**0.001**
*PnKTI B1*	0.710	**0.001**
*PnKTI B5*	0.597	**0.009**
*PnKTI D2*	0.582	**0.011**

## Discussion

Here we describe sequence analyses and expression patterns of Kunitz-type trypsin inhibitors (KTIs) in black poplar (*Populus nigra*), including ten full-length cDNA sequences, of which six had not been described before in *P. nigra*. Eight of these PnKTIs were studied in the context of herbivore species-specific induction patterns in leaves and we could show that beetle herbivory elicits a much stronger transcriptional response than caterpillar herbivory of the same magnitude.

### Expression levels and inducibility of individual black poplar KTI genes

The up-regulation of protease inhibitor (PI) transcription and activity has been described previously, also in black poplar [27, 38]. However, both constitutive expression levels and amplitude of induction vary between studies. In our study, members of the C-subfamily generally showed low expression levels and little or no up-regulation upon herbivory (Fig. 2). In contrast, Ma et al. [27] observed stronger herbivore induction for most of the genes in this subfamily. This suggests that the regulation of *KTI* transcription depends on more factors than herbivore feeding or wounding alone. Certain traits of the plants, such as age, genotype [44] or previously experienced damage may play a role, but also the experimental conditions such as abiotic conditions, timing [27, 39] or damage severity could potentially influence expression levels. However, there are also consistent patterns among the different studies. In our study, *PnKTI D2*, the only member of the D subfamily, showed the highest inducibility, i.e. relative change upon herbivory (Fig. 4). The same gene was amongst the most up-regulated *KTI*s upon herbivory and mechanical wounding in another black poplar study [27]. Similarly, the high herbivore-induced expression levels of *PnKTI A14* in our experiments (Fig. 2; Table S5) match well with the results obtained for the corresponding ortholog in a hybrid poplar species (*P. trichocarpa* x *deltoides*) after herbivory and mechanical wounding [39]. However, this gene also showed relatively high transcript abundance in undamaged controls, assuming also a role in constitutive defense or primary metabolism. On the contrary, *PnKTI D2*, which displays minimal expression levels in undamaged tissue in our and a similar study [27], seems to act exclusively in induced anti-herbivore defense.

There was no correlation between gene expression for most of the *PnKTI* genes and damage severity, which suggests a threshold-based activation of *PnKTI* transcription rather than continuous control, in which more damage would lead to higher *KTI* transcript levels. Furthermore, we found a strong positive relationship between the trypsin-inhibiting activity in poplar leaves and the transcription levels for all *PnKTI* genes. This indicates that *P. nigra* KTI activity is predominantly controlled at the transcriptional level and hence by de novo biosynthesis. The importance of de novo biosynthesis of stress-induced PIs has already been demonstrated in rice [40].

### Herbivore specificity in PnKTI induction

When we analyzed the transcription of *KTI*s in leaves damaged by different insect herbivores, it became evident that beetles elicited a much stronger induction of all tested *KTI*s than caterpillars (Fig. 4). Similar observations come from pine trees [35] and milkweed [1, 48], where beetle herbivory induced stronger defense responses (resins and terpenes, or latex, respectively) compared to caterpillar herbivory. Species-specificity has been reported for the induction of PIs in other systems, though not in poplar trees. In soybean, damage by fall armyworm caterpillars increased the activity of PIs, whereas thrips damage did not [43]. De Oliveira et al. [11] even observed varying response of tomato PIs to damage by herbivores of the same genus. They showed that PI activity was induced by the spider mite *Tetranychus urticae*, but was suppressed by *T. evansi* [11]. Interestingly, feeding damage by lepidopteran and coleopteran herbivores in tomato yielded opposite results to our study in black poplar. Here, gene expression and trypsin inhibiting activity was more strongly induced by the tobacco hornworm than by the Colorado potato beetle [10].

The difference in *PnKTI* expression between beetle (*Phratora vulgatissima*) and caterpillar (*Lymantria dispar* and *Amata mogadorensis*) herbivory might be based on the different damage pattern these insects cause, even though all three of them are leaf chewers and removed the same total leaf area. While caterpillars removed large chunks of the leaves, the beetles caused small but numerous lesions in the leaves (Fig. 1). The number of lesions was found to be a key factor determining the emission of volatiles, another important anti-herbivore defense trait in black poplar [31]. Other factors, such as the duration of damage or the chemical compounds deposited on the plant may also be important. When artificial damage was administered to lima bean with a mechanical caterpillar, changes in the amount of time that damage lasted as well as the area damaged affected the emission of volatiles [34]. Furthermore, species-specific compounds in the saliva could trigger distinct defense responses or the magnitude of response as reported here. The importance of insect-derived elicitors for PI induction has been demonstrated in another poplar species, where mechanical wounding and simultaneous application of oral secretions from forest tent caterpillars suppressed the induction of PIs [39]. It is likely that oral secretions of the insects used in this study also exhibit a suppressive effect, maybe with varying efficacy on PI induction. Whether herbivore host range, comparing generalists such as *L. dispar* and *A. mogadorensis* versus specialists such as *P. vulgatissima*, plays a role in the induction of PIs, is not clear. Specialists usually possess a higher tolerance towards specific chemical defenses of their hosts, such as salicinoids in black poplar trees [3]. An increased induction of a defense, such as the PIs, to specialist herbivores could therefore be a more effective way to defend against these insects. Future studies using more herbivore species, or generalists and specialists that are more closely related to each other and cause similar feeding patterns, are necessary to determine if herbivore host range influences PI induction.

Whether the herbivore specific induction patterns of *PnKTI*s have ecological relevance is another open question. One factor that plays an important role in this context is ‘effect specificity’ [20]. PIs possess varying effectiveness in defense against different herbivores, as could be observed in the performance of five different herbivores that had been reared on PI-supplemented diets [8]. Similarly, the cotton bollworm exhibited distinct preference and performance towards different classes of protease inhibitors [25]. This can be explained by the fact that PIs, on the one hand, vary in their ability to inhibit different proteases, i.e. trypsin, chymotrypsin and elastase [29], and that insects, on the other hand, vary in their gut protease activities [8, 20]. Additionally, the gut pH, which differs substantially between Lepidoptera and Coleoptera [20], also influences the inhibitory activity of PIs [49]. It would therefore be interesting to dissect the role of individual KTIs in black poplar towards different insect herbivores, for example by using transgenic trees or diet supplementation of recombinant KTIs. ‘Response specificity’ towards herbivore species is believed to be more cost-effective for a plant than a similar response to all herbivores [20]. Keeping in mind the fitness costs that are linked to the biosynthesis of PIs [18], a plant might aim to induce a subset of PIs to which a herbivore is most sensitive. In this context, PI activity should not be evaluated independently of other plant defense compounds. In tobacco, PIs function synergistically with the chemical defense compound nicotine, which becomes more toxic when herbivores have to compensate for nutritional deficits by increased feeding activity [45]. Black poplar contains toxic defense compounds called salicinoids, which have been shown to negatively influence herbivore performance and survival [4, 5]. Therefore, possible synergistic effects between salicinoids and PIs, the two main components of direct defense in this tree should be investigated in future studies.

## Conclusion

Our major conclusion is that PI induction in black poplar leaves depends on the identity of the feeding herbivore, with beetles inducing a stronger response than caterpillars. Furthermore, PI activity is regulated at the level of transcription and most likely in a threshold-based fashion. However, most of the molecular mechanisms underlying the patterns observed and their ecological consequences remain to be elucidated.

## Supplementary Information

**Additional file 1: Table S1**. Feeding damage by the three herbivores and trypsin-inhibiting activity in poplar. **Table S2**. Primer sequences used for cloning and qRT-PCR. **Table S3**. Differential expression of contigs annotated as protease inhibitors in the transcriptome of black poplar leaves. **Table S4**. Nomenclature of KTI homologs in this and other studies. **Table S5**. Quantification cycles of the qRT-PCR analysis for individual *KTI* genes.

## Data Availability

The short-read data have been deposited in the EBI short read archive (SRA) with the following sample accession numbers: ERS5844847- ERS5844862. The complete study can also be accessed directly using the following URL: http://www.ebi.ac.uk/ena/data/view/PRJEB43369.

## Abbreviations

KTI: Kunitz-type trypsin inhibitors
qRT-PCR: Quantitative real-time PCR
cDNA: Complementary DNA
PI: Protease inhibitor
HAMPs: Herbivore-associated molecular patterns
DAMPs: Damage-associated molecular patterns
ORF: Open reading frame
PCR: Polymerase chain reaction
MANOVA: Multivariate analysis of variance
