# Supplementary material for: Poplar protease inhibitor expression differs in an herbivore specific manner
Source: BMC Plant Biol. 2021 Apr 9;21:170. doi: 10.1186/s12870-021-02936-4 (PMC8033671; doi:10.1186/s12870-021-02936-4)
Supplement: Supplementary file 1 — Additional file 1: Table S1. Feeding damage by the three herbivores and trypsin-inhibiting activity in poplar. Table S2. Primer sequences used for cloning and qRT-PCR. Table S3. Differential expression of contigs annotated as protease inhibitors in the transcriptome of black poplar leaves. Table S4. Nomenclature of KTI homologs in this and other studies. Table S5. Quantification cycles of the qRT-PCR analysis for individual KTI genes. [file 12870_2021_2936_MOESM1_ESM.pdf]

Supplementary Material:

Title: Poplar protease inhibitor expression differs in an herbivore specific manner

Authors: Franziska Eberl[1]*, Thomas Fabisch[1], Katrin Luck[1], Tobias G. Köllner[1], Heiko Vogel[2], Jonathan Gershenzon[1], Sybille B. Unsicker[1]

[1] Max Planck Institute for Chemical Ecology (MPI-CE), Department of Biochemistry, Hans-Knöll-Str. 8, 07745 Jena

[2] MPI-CE, Department of Entomology, Hans-Knöll-Str. 8, 07745 Jena

*Corresponding author: Email: feberl@ice.mpg.de; Phone: +49 (0)3641 57 1337

Table S1. Feeding damage by the three herbivores and trypsin-inhibiting activity in poplar.

Table S2. Primer sequences used for cloning and qRT-PCR.

Table S3. Differential expression of contigs annotated as protease inhibitors in the transcriptome of black poplar leaves.

Table S4. Nomenclature of KTI homologs in this and other studies.

Table S5. Quantification cycles of the qRT-PCR analysis for individual *KTI* genes.

Table S1. Feeding damage on black poplar leaves after 2 d of herbivore feeding by *Lymantria dispar*, *Amata mogadorensis* and *Phratora vulgatissima* and trypsin-inhibiting activity in these leaves. Shown is the leaf area loss (% of total leaf area) and inhibitory activity (equivalents to soybean trypsin inhibitor (STI), µg STI g^{-1} dry weight (DW)) as mean ± SEM (n = 6). The three groups were not found to be significantly different (ANOVA: $F_{(2)}$ = 0.762; P = 0.484). Data are taken from Fabisch *et al.* (2019), and the parameters re-calculated for the six replicates selected for this study.

**Herbivore**	**Damage (%)**	**KTI activity (µg g^{-1} DW)**
L. dispar	19.0 ± 5.7	95.7 ± 12.9
A. mogadorensis	12.3 ± 4.8	93.1 ± 26.4
P. vulgatissima	12.0 ± 1.6	257.6 ± 23.2

Table S2. Sequences of primers used for full-length cloning (FL) and gene expression analysis via quantitative real-time PCR (qRT-PCR). Primer sequences for *Actin* were taken from Ramiraz-Carvajal*et al.* 2008[1].

Usage	Name	Forward primer (5' → 3')	Reverse primer (5' → 3')
FL	SQ16949	ATGAAGATCACTAACGTTCTAG	CTATTCATCTGGTTCAATCATAAC
FL	SQ33325 (a)*	CACCCATGTGTCTGATGTGG	TCAATATGCATCTGGTTCG
	SQ33325 (b)*	ATGGAGATCACTAAATTTCTAGG	GTATCCTTCCGTTGTTGGACT
FL	SQ33325-2	ATGGAGATCACTAAATTTCTAGG	TCAATATGCATCTGGTTCG
FL	SQ14956	ATGAAGATCACTAAATTTCTAGGG	TTATGTGCTCTCAATGCG
FL	SQ36485	ATGAAGATCACCAAGTTTGT	TCAGGAGCTTTTATCTGC
FL	SQ2215	ATGAAGATCTCTAACTTTCTAGTG	TTACACCATTTTATACTCTATTTTAGA
FL	SQ1996	ATGAAGATTACTAACTTTCTAGTGC	TTACATCATTTTATACTCTATTTTAGAAGA
FL	VP33729	ATGAAGTCTACATTGTTGGT	TCATATGGATGAACTTAAAGGC
FL	SQ6530	ATGAAGTCTACATTGTTGGT	TCATATGGATGAACTTAAAGGC
FL	SQ287	ATGAAGAATATTATGTTACTACCCC	TTACACAACAGCTTTTAATCC
qRT-PCR	SQ2215	TTACTGTCTCCAATGAGCCATG	ATGAGCAGATGGGTTCGG
qRT-PCR	SQ287	GCGTTACAGGTACACCAG	ACAAATGAGCCTCCCACG
qRT-PCR	SQ36485	ACAACTCTTGCGGTCTCTG	CATTGTTGGCCTCAACTCC
qRT-PCR	SQ6530	TTCGGACCTGTTACAAGGC	GAAACTAGCTTGTACCCTATGC
qRT-PCR	SQ1580	GTTGTGTTTTCTCCAATGAGCG	GGACGCATGAGCATTCACAT
qRT-PCR	VP33729	CTAATATGCCAGCCTTCTC	GTTGGACCAGTTACAAGGT
qRT-PCR	SQ34879	CACCCATGTGTCTGATGTG	GAAGAGGGCCGACATTGG
qRT-PCR	SQ8996	GCGCAACCGGTTTAAACC	GATTCAATTCAACAGCCAAAG
qRT-PCR	Actin	CCCATTGAGCACGGTATTGT	TACGACCACTGGCATACAGG

[*] two primer pairs were used to amplify the gene in two parts. [1] Ramirez-Carvajal GA, Morse AM, Davis JM. 2008. *New Phytologist* **177:**77–89.

Table S3. Contigs in the transcriptome of black poplar leaves damaged by *L. dispar* and *P. vulgatissima* that were annotated as protease inhibitors, and their differential expression (ratio of RPKM values) compared to the respective undamaged control treatments (Ctrl 1 and 2). Contigs are sorted by their regulation pattern (up-regulated, differentially regulated, down-regulated upon herbivory) and further by the *P*-value of 'Ctrl 1 vs. *L. dispar*' in descending significance. Annotations including 'Kunitz' are marked in bold; for these contigs, the gene names used in this study are given (PnKTI).

**Name**			**Ctrl 1 vs. *L. dispar***		**Ctrl 2 vs. *P. vulgatissima***	
**ID**	**PnKTI**	**Sequence Annotation**	**Diff. expr.**	***P***	**Diff. expr.**	***P***
Up-regulated upon herbivory						
SQ1996	*PnKTI A6*	**Kunitz trypsin inhibitor ti3**	4.511 up	0.00193	7.782 up	0.0000667
SQ287	*PnKTI D2*	**Kunitz trypsin inhibitor**	1547.915 up	0.00318	251.880 up	0.00045
SQ14956	*PnKTI A14*	**Kunitz trypsin inhibitor**	1340.240 up	0.00828	3121.864 up	0.0000843
SQ6530	*PnKTI B5*	**Kunitz trypsin inhibitor**	139.616 up	0.00852	127.805 up	0.00505
SQ36485	*PnKTI A7*	**Kunitz-type protease inhibitor kpi-**	1512.845 up	0.00916	2087.716 up	0.0000093
SQ27194	*PnKTI A12*	**Kunitz trypsin inhibitor 3**	12.698 up	0.0105	2.847 up	0.0288
SQ8430		inter-alpha-trypsin inhibitor heavy	5.710 up	0.0138	3.448 up	0.0242
SQ16949	*PnKTI A2*	**Kunitz-type protease inhibitor kpi-**	1609.628 up	0.0141	2529.060 up	0.0000375
SQ37196		inter-alpha-trypsin inhibitor heavy	5.611 up	0.0146	3.428 up	0.0113
SQ17376		inter-alpha-trypsin inhibitor heavy	6.847 up	0.0152	4.168 up	0.012
SQ8431		inter-alpha-trypsin inhibitor heavy	4.228 up	0.0165	2.870 up	0.00411
SQ24859		inter-alpha-trypsin inhibitor heavy	5.591 up	0.0218	2.972 up	0.0166
SQ2215	*PnKTI A15*	**Kunitz trypsin inhibitor ti3**	40.816 up	0.0229	72.350 up	0.000316
SQ33325	*PnKTI A13*	**Kunitz-type protease inhibitor KPI-**	24381.159 up	0.0247	7431.743 up	0.00283
SQ49470		inter-alpha-trypsin inhibitor heavy	8.483 up	0.0832	4.155 up	0.065
SQ24955	*PnKTI B2*	**Kunitz trypsin protein inhibitor 3**	38.630 up	0.188	3.239 up	0.277
SQ43060	*PnKTI C1*	**truncated Kunitz trypsin inhibitor family protein**	40.465 up	0.192	58.511 up	0.0764
SQ6918		protease inhibitor seed storage lipid transfer family protein	2.266 up	0.227	1.814 up	0.00185
SQ61412		protease inhibitor seed storage lipid transfer family protein	14.550 up	0.392	153.666 up	0.0619
VP33729	*PnKTI B1*	**Kunitz trypsin inhibitor 4**	114,626 up	0,409	na	na
SQ47062		inter-alpha-trypsin inhibitor heavy	2.573 up	0.441	2.334 up	0.584
SQ49510	*PnKTI C3*	**truncated Kunitz trypsin inhibitor family protein**	14.149 up	0.458	40.486 up	0.228
SQ22824		protease inhibitor seed storage lipid transfer family protein	7.775 up	0.489	1.674 up	0.344
SQ10660		protease inhibitor seed storage lipid transfer family protein	1.874 up	0.507	1.953 up	0.178

Name			Ctrl 1 vs. *L. dispar*		Ctrl 2 vs. *P. vulgatissima*	
**ID**	**PnKTI**	**Sequence Annotation**	**Diff. expr.**	***P***	**Diff. expr.**	***P***
SQ23804	*PnKTI C6*	**truncated Kunitz trypsin inhibitor family protein**	7.688 up	0.549	3.869 up	0.735
SQ172	*PnKTI C7*	**truncated Kunitz trypsin inhibitor family protein**	1.778 up	0.552	12.017 up	0.0805
SQ56957	*PnKTI C5*	**truncated Kunitz trypsin inhibitor family protein**	4.550 up	0.747	3.794 up	0.765
SQ21088		protease inhibitor seed storage lipid transfer family protein	1.224 up	0.875	1.398 up	0.539
SQ33797		serine protease inhibitor	1.229 up	1	3.318 up	0.785
Differentially regulated upon herbivory						
SQ29472		serine-type endopeptidase inhibitor	108.536 down	0.00000369	4.379 up	0.756
SQ47318		cysteine proteinase inhibitor 12-like	1.434 down	0.205	1.439 up	0.633
SQ10062		cysteine protease inhibitor	1.306 up	0.324	1.004 down	1
SQ26391		protease inhibitor seed storage lipid transfer family protein	1.527 up	0.858	1.583 down	0.608
SQ17957		inter-alpha-trypsin inhibitor heavy chain-related family protein	1.090 down	0.911	1.231 down	0.658
SQ12658		cysteine proteinase inhibitor	1.001 up	1	1.176 down	0.756
SQ18761		cysteine proteinase inhibitor	1.046 up	1	1.031 down	1
Down-regulated upon herbivory						
SQ64031		cysteine proteinase inhibitor b-like	5.168 down	0.0282	3.143 down	0.0767
SQ7576		cysteine proteinase inhibitor 12-like	1.318 down	0.0655	1.923 down	0.103
SQ35734		subtilisin inhibitor	1.744 down	0.142	2.001 down	0.168
SQ7450		cysteine proteinase inhibitor	1.596 down	0.22	1.370 down	0.214
SQ52953		cysteine proteinase inhibitor 12-like	1.232 down	0.509	1.259 down	0.663
SQ7577		cysteine inhibitor 1	1.140 down	0.6	1.262 down	0.174
SQ34850		inhibitor of trypsin and hageman factor-like protein	1.710 down	0.715	1.666 down	0.658
SQ17958		inter-alpha-trypsin inhibitor heavy chain-related family protein	1.058 down	0.851	1.095 down	0.68

na - not available; this contig and its expression was taken from another transcriptome of the same *P. nigra* genotype (unpublished) with comparable *L. dispar*, but not *P. vulgatissima* herbivory treatment.

Table S4. Nomenclature of Kunitz-type protease inhibitors reported in this study and their corresponding homologs as Potri-IDs from the *Populus trichocarpa* genome v3.0 and genes described in other studies. Similarity values (% sim) show the percentage of identical base pairs between the respective sequences with those published in our study based on their full-length open reading frame, unless stated otherwise.

**Eberl *et al.* 2020**	***P. trichocarpa* genome**		**Ma *et al.* 2011 [1]**		**Philippe *et al.* 2009 [2]**		**Other studies**	
**Name (*P. nigra*)**	**Potri-ID**	**% sim**	**Name (*P. nigra*)**	**% sim**	**Name (*P. spp*)**	**% sim**	**Name (*P. spp*)**	**% sim**
PnKTI A2	Potri.010G007800.1	98.5	-	-	PtxnKPI-A2	98.5	TI6[3]	97.7
PnKTI A4	Potri.010G007900.1	99.3	-	-	PtxnKPI-A5/ PtxdKPI-A5	99.3/ 99.2		
PnKTI A6	Potri.019G124400.1	97.7	PnKTI A6	98.0	PtxnKPI-C6.1/ PtxdKPI-C7*	99.5/ 99.5	TI3[3]	98.0
PnKTI A7	Potri.019G121900.1	100	PnKTI A7	99.5	PtxdKPI-C2.1	99.5		
PnKTI A12	Potri.003G097900.2	100	PnKTI A12	99.2	PtiKPI-2	99.2		
PnKTI A13	-	-	-	-	-	-		
PnKTI A14	Potri.T029200.1*	92.2	-	-	PtxdKPI-B5*/ PtxnKPI-B7*	98.3/ 97.1	GWIN 3[4]; PnTIH1.1[5]	98.5/100
PnKTI A15	-	-	-	-	-	-		
PnKTI B1	Potri.004G067800.1	99.7	PnKTI B1	99.7	PtiKPI-D1.2	99.7	TI4[3]	99.7
PnKTI B2	Potri.004G067900.1	99.1	PnKTI B2	98.9	PtxdKPI-D2	99.1		
PnKTI B5	-	-	-	-	PtxnKPI-D8	100		
PnKTI C1	Potri.001G309900.1	97.2	PnKTI C1	99.7	-	-		
PnKTI C3	Potri.007G111600.1	99.2	PnKTI C3	99.2	-	-		
PnKTI C5	Potri.004G000400.1	99.2	PnKTI C5	99.2	PtxnKPI-F4	99.3		
PnKTI C6	Potri.019G011000.1	98.8	PnKTI C6	100	-	-		
PnKTI C7	Potri.007G111800.1	96.6	PnKTI C7	96.7	PtxdKPI-F9	97.4		
PnKTI D2	Potri.019G088200.1	97.9	PnKTI D2*	99.2	PtxdKPI-E1	99.4	TI5[4]	99.2

[1] Ma Y, Zhao Q, Lu M-Z, Wang J (2011). *Tree Genetics & Genomes*, **7:** 431-441.[2] Philippe RN, Ralph SG, Külheim C, Jancsik SI, Bohlmann J (2009). *New Phytologist*, **184:** 865-884. [3] Major IT &Constabel CP (2008). *Plant Physiology*, **146:** 888-903.[4]Bradshaw HD, Hollick JB, Parsons TJ, Clarke HRG, Gordon MP. *Plant Molecular Biology*, **14:** 51-59. [5] Nishiguchi M, Yoshida K, Sumizono T, Tazaki K (2002). *Molecular Genetics and Genomics*, **4:** 506-514. * incomplete ORF.

Table S5. C_q (quantification cycle) values of the qRT-PCR analysis for individual *KTI* genes in black poplar leaves without damage (Control) or after herbivory by lepidopteran caterpillars (*L. dispar, A. mogadorensis*) or adult beetles (*P. vulgatissima*). Shown are means ± SEM for each treatment group (n = 6).

**Gene**	**Control**	***L. dispar***	***A. mogadorensis***	***P. vulgatissima***
PnKTI A6	23 ± 0.3	19 ± 0.4	19 ± 0.7	17 ± 0.3
PnKTI A7	31 ± 0.6	27 ± 1.0	28 ± 0.6	26 ± 0.5
PnKTI A13	31 ± 0.5	20 ± 0.8	21 ± 0.8	17 ± 0.4
PnKTI A14	27 ± 0.5	17 ± 0.8	17 ± 0.7	14 ± 0.4
PnKTI A15	25 ± 0.3	18 ± 0.5	18 ± 0.5	16 ± 0.2
PnKTI B1	33 ± 0.7	27 ± 0.8	27 ± 0.8	23 ± 0.8
PnKTI B5	36 ± 0.9	32 ± 0.6	31 ± 0.7	28 ± 0.5
PnKTI D2	35 ± 0.4	25 ± 0.8	23 ± 0.8	20 ± 0.6
Actin*	20 ± 0.4	20 ± 0.2	20 ± 0.4	20 ± 0.2

* house-keeping gene for normalization
